# Second-line chemotherapy for patients with advanced gastric cancer: who may benefit?

**DOI:** 10.1038/sj.bjc.6604732

**Published:** 2008-10-28

**Authors:** V Catalano, F Graziano, D Santini, S D'Emidio, A M Baldelli, D Rossi, B Vincenzi, P Giordani, P Alessandroni, E Testa, G Tonini, G Catalano

**Affiliations:** 1 Department of Medical Oncology, Azienda Ospedaliera ‘Ospedale San Salvatore’, Pesaro, Italy; 2 Department of Medical Oncology, Università Campus Bio-Medico, Roma, Italy; 3 Data Management, Department of OncoHematology, Azienda Ospedaliera ‘Ospedale San Salvatore’, Pesaro, Italy; 4 Department of Medical Oncology, Hospital of Urbino, Urbino, Italy

**Keywords:** second-line chemotherapy, prognostic factor, metastatic gastric cancer

## Abstract

No established second-line chemotherapy is available for patients with advanced gastric cancer failing to respond or progressing to first-line chemotherapy. However, 20–40% of these patients commonly receive second-line chemotherapy. We evaluated the influence of clinico-pathologic factors on the survival of 175 advanced gastric cancer patients, who received second-line chemotherapy at three oncology departments. Univariate and multivariate analyses found five factors which were independently associated with poor overall survival: performance status 2 (hazard ratio (HR), 1.79; 95% CI, 1.16–2.77; *P*=0.008), haemoglobin ⩽11.5 g l^−1^ (HR, 1.48; 95% CI, 1.06–2.05; *P*=0.019), CEA level >50 ng ml^−1^ (HR, 1.86; 95% CI, 1.21–2.88; *P*=0.004), the presence of greater than or equal to three metastatic sites of disease (HR, 1.72; 95% CI, 1.16–2.53; *P*=0.006), and time-to-progression under first-line chemotherapy ⩽6 months (HR, 1.97; 95% CI, 1.39–2.80; *P*<0.0001). A prognostic index was constructed dividing patients into low- (no risk factor), intermediate- (one to two risk factors), or high- (three to five risk factors) risk groups, and median survival times for each group were 12.7 months, 7.1 months, and 3.3 months, respectively (*P*<0.001). In the absence of data deriving from randomised trials, this analysis suggests that some easily available clinical factors may help to select patients with advanced gastric cancer who could derive more benefit from second-line chemotherapy.

Despite a declining incidence in many developed countries, gastric cancer remains the second most common cause of cancer deaths, and it is responsible for about 12% of all cancer-related deaths worldwide (Parkin *et al*, 1999). As for most gastrointestinal cancers, the management of gastric cancer is based on the surgical resection of the primary tumour. However, more than two-thirds of patients diagnosed with gastric cancer will have unresectable disease (Parkin *et al*, 1999; Catalano *et al*, 2005; Macdonald, 2006), and despite the fact that surgical pathological R0 resection can be curative for many patients, recurrence rates are about 70% and 5-year survival rate is lower than 30% (Catalano *et al*, 2005). For patients with unresectable disease, or developing recurrent disease after curative resection, evidence supports the use of palliative chemotherapy with the aims of improving symptoms, quality of life, and possibly prolonging survival (Murad *et al*, 1993; Pyrhonen *et al*, 1995; Glimelius *et al*, 1997). Several chemotherapy agents are considered active in advanced gastric cancer. Over the past decades, many anticancer drugs have been investigated, such as 5-fluorouracil (5-FU), cisplatin, anthracyclines, oral fluoropyrimidines, irinotecan, oxaliplatin, and docetaxel (Catalano *et al*, 2005; Dank *et al*, 2005; Al-Batran *et al*, 2006; Kang *et al*, 2006; Van Cutsem *et al*, 2006; Cunningham *et al*, 2008). Combination chemotherapy regimens have been developed in the hopes of improving response rate and overall survival (OS). Unfortunately, the benefits of combination chemotherapy have been modest (Wagner *et al*, 2006). In general, regimens containing 5-FU and cisplatin are widely accepted as potential standard therapies with a response rate of 25–45% and median OS times of 7–9 months (Ajani 2005; Catalano *et al*, 2005; Van Cutsem *et al*, 2006; Cunningham *et al*, 2008). Although a large proportion of patients with metastatic or recurrent gastric cancer may initially respond to chemotherapy, they ultimately progress. In addition, many patients have primary refractory disease. The median survival at progression after first-line chemotherapy for metastatic gastric cancer is about 2.5 months.

Yet, there is no established second-line chemotherapy for gastric cancer, but it is used in 20–40% of patients (Chau *et al*, 2004a; Lee *et al*, 2007). In three phase III randomised trials of 5-FU-based first-line therapy enrolling a total of 1080 patients, 20% of them went on to receive second-line chemotherapy, with a response rate observed as 13.3% (95% CI, 6.8–22.5%), and a median survival from starting second-line therapy of 5.6 months (Chau *et al*, 2004a).

During the past few decades, many phase II reports of second-line therapy including gastric cancer patients have been published, but no randomised phase III trial investigating second-line chemotherapy compared with best supportive care is available. There is a great heterogeneity within each of phase II trial, partly because of the variability in the responsiveness to first-line chemotherapy, and the nature of previous chemotherapy (cisplatin-based or not). Morevover, available data have limitations because of a publication bias; trials generally include small numbers of participants and they do not support evidence for identifying patients who are more likely to benefit from second-line chemotherapy (Wilson *et al*, 2005).

A decision whether starting or not second-line chemotherapy for an individual patient may be a common clinical scenario. Several factors should be considered: the potential cumulative toxicity, especially for those patients with low performance status (PS), the extent of disease, the need of active drugs, the lack of cross-resistance to drugs previously used. But second-line therapy could not be appropriate for all patients, and prediction of treatment outcome may allow for the identification of patients who would derive very little benefit from second-line chemotherapy. The aim of this analysis was to explore clinico-pathologic factors that may be associated with survival of patients treated with second-line chemotherapy. In the absence of definitive phase III trials, consideration of some of these factors, if any, may assist in the selection of patients for further treatment. However, as this analysis was performed on patients receiving cytotoxic drugs, it is not possible to extrapolate results for patients treated with novel biological agents and within clinical trials.

## Patients and methods

From January 1995 to August 2006, 625 patients received first-line chemotherapy at three oncology departments (San Salvatore Hospital, Pesaro; Hospital of Urbino; Campus Biomedico, Rome). Of them, 175 (28%) consecutive patients received second-line chemotherapy for locally advanced or metastatic gastric cancer. No patient receiving second-line chemotherapy was excluded from this analysis. The criteria for case inclusion were as follows: (i) histologically confirmed diagnosis of gastric cancer; (ii) previous treatment with first-line chemotherapy, given until disease progression, unacceptable toxicity, or patient's refusal; (iii) presence of measurable disease; (iv) progressing disease after the first-line chemotherapy; (v) availability of clinico-pathological data at the beginning of second-line chemotherapy.

Approval of the study was obtained from the local research and ethics committee.

The clinical tumour response was assessed according to Response Evaluation Criteria in Solid Tumors (RECIST) (Therasse *et al*, 2000).

Factors included in the univariate analyses were as follows: age, sex, PS, loss of weight >5%; haemoglobin, serum albumin, CEA levels; previous gastrectomy, Lauren classification, tumour grade, tumour location, metastasis to liver, peritoneum, number of metastatic sites; time-to-progression (TTP), objective response to first-line chemotherapy, regimen used as first-line chemotherapy (oxaliplatin or cisplatin plus 5-FU-based *vs* 5-FU-based). Laboratory variables were initially recorded as continuous variables and later dichotomised according to the median value of each variable. Performance status was evaluated according to the Eastern Cooperative Oncology Group (ECOG) criteria. Time-to-progression under first-line chemotherapy was measured from the date of the beginning of the treatment to the date of progression. To refine its possible association with survival to second-line chemotherapy, TTP under first-line chemotherapy was studied dichotomising the study population according to disease progression less than or equal to the median TTP *vs* disease progression greater than the median TTP.

### Statistical analysis

This is a multicentric, retrospective study. The primary end point of the study was OS. Overall survival was measured from the date of the first cycle of second-line chemotherapy to the date of death or the last follow-up visit. Survival data were analysed using the Kaplan–Meier product-limit method (Kaplan and Meier, 1958). Comparison of survival curves were performed using log-rank test (Peto and Peto, 1972). A prognostic model was established by searching all variables that significantly influenced OS at a level of *P*-values <0.05 in the univariate analysis. Multivariate analysis was carried out using stepwise Cox proportional hazards regression modelling. *P*-values <0.05 were considered statistically significant and all *P*-values correspond to two-sided significance tests.

## Results

The characteristics of patients included in this analysis are shown in Table 1. The median age was 64 years with a range of 38–83 years. Sixty-six percent of patients were male, and 23.4% had ECOG PS 2. Only four patients had locally advanced disease, and 77.7% of patients received previous gastrectomy.

### Treatment

First-line chemotherapy contained 5-FU (*n*=13), 5-FU and cisplatin (*n*=141), or 5-FU and oxaliplatin (*n*=21) (Table 2). Twelve patients (6.8%) experienced a complete response, and 71 patients (40.6%) a partial response for an overall response rate of 47.4% (95% CI, 40.0–54.8). Median TTP under first-line chemotherapy was 6 months (range, 1–68 months).

All the patients received 5-FU as second-line chemotherapy, given as bolus i.v. or continuous infusion and combined with other drugs. Second-line chemotherapy consisted of regimens containing 5-FU and cisplatin (*n*=21), 5-FU and oxaliplatin (*n*=31), 5-FU and irinotecan (*n*=51), 5-FU and paclitaxel or docetaxel (*n*=25), and 5-FU-based (*n*=47). In any case, no combination of drugs received as first-line treatment was subsequently given at disease progression. A complete response to second-line chemotherapy was achieved in 3 patients and a partial response in 25 patients, for an overall response rate of 16.0% (95% CI, 10.6–21.4).

### Univariate and multivariate analysis

**Figure 1:**
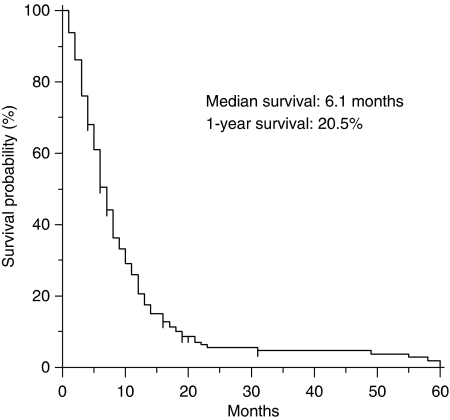
Overall survival curve for the whole group (*n*=175).

At the time of analysis, 163 (93.1%) patients had died from tumour progression. Median survival for the whole group was 6.1 months, and 1-year OS was 20.5% (95% CI, 14.4–26.6) (Figure 1).

At univariate analysis (Table 3), eight variables were significantly associated with poor survival time: ECOG PS 2, loss of weight >5%, haemoglobin ⩽11.5 g l^−1^, CEA >50 ng ml^−1^, the presence of peritoneal carcinomatosis, a number of metastatic sites 3–4, TTP under first-line chemotherapy ⩽6 months, and previous chemotherapy with regimens containing 5-FU alone. No statistically significative difference was found between each regimen used as second-line chemotherapy.

Multivariate regression analysis (Table 4) included the eight variables that were found to have prognostic significance in univariate analysis. These features were available for all 175 patients. Five factors, PS, haemoglobin, CEA, the number of metastatic sites, and TTP under first-line chemotherapy, showed independent prognostic role.

The multivariate analysis was also performed by stratificating for the second-line chemotherapy received. However, we found no interaction between any second-line treatment and each clinico-pathologic variable (data not shown).

**Figure 2:**
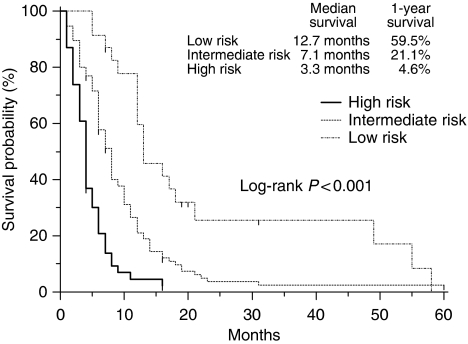
Survival curves according to the risk groups. Marks on survival curves denote censored observations.

Then, a multivariate prognostic model was constructed by incorporating all the five adverse prognostic factors. The prognostic grouping was carried out according to the following criteria: low-risk group, patients with no prognostic factor (*n*=25); intermediate-risk group, patients with one or two negative prognostic factors (*n*=101); high-risk group, patients with three to five negative prognostic factors (*n*=49). The survival curves according to the prognostic model are shown in Figure 2. Median survival times for low-, intermediate-, and high-risk groups were 12.7, 7.1, and 3.3 months, respectively. One-year survival rates for each group were 59.5% (95% CI, 39.0–79.9), 21.1% (95% CI, 12.7–29.5), and 4.6% (95% CI, 0–10.8), respectively. There were marked significant survival differences among the three risk groups (*P*<0.001).

When compared to the low-risk group, the intermediate-risk group had a two-fold (HR, 2.18; 95% CI, 1.38–3.30), and the high-risk group had a 3.5-fold (HR, 3.61; 95% CI, 3.4–11.67) increased risk of death.

## Discussion

Despite new schedules and association of drugs, patients with advanced gastric cancer treated with first-line chemotherapy have median OS rarely approaching 11 months (Catalano *et al*, 2005; Dank *et al*, 2005; Al-Batran *et al*, 2006; Kang *et al* 2006; Van Cutsem *et al*, 2006; Cunningham *et al*, 2008). No significant steps ahead have been moved since the past decade. Many patients did not respond to first-line chemotherapy or have progression of their disease within some months from the end of treatment.

To improve survival of advanced gastric cancer patients, more effective first-line regimens are necessary. No established second-line therapy is available and decision whether to treat or not such patients with second-line chemotherapy is crucial.

This analysis based on the individual data of 175 consecutive patients treated with second-line chemotherapy identifies five independent prognostic factors: PS, haemoglobin level, CEA level, number of metastatic sites, TTP under first-line chemotherapy. To our knowledge, this is the first report, which assess pre-treatment clinical prognostic factors for patients receiving second-line therapy for advanced gastric cancer. Conversely, three different studies (Yoshida *et al*, 2004; Chau *et al*, 2004b; Lee *et al*, 2007) aimed to identify clinical prognostic factors for metastatic gastric cancer patients undergoing first-line chemotherapy. Chau *et al* (2004b) assessed prognostic factors on 1080 patients with locally advanced and metastatic oesophago-gastric cancer. Four independent poor prognostic factors were identified by multivariate analysis: PS ⩾2, liver metastases, peritoneal metastases, and alkaline phosphatase ⩾100 U l^−1^. A prognostic index was developed dividing patients into good (no risk factor), moderate (one to two risk factors) or poor (three to four risk factors) risk groups. One-year survival for good, moderate, and poor risk groups were 48.5, 25.7, and 11%, respectively, with a highly significant difference among the three groups (*P*<0.0001). In the second study (Yoshida *et al*, 2004), the Japan Clinical Oncology Group (JCOG) showed that better PS, a limited number of metastatic sites, and macroscopically scirrhous type tumours were significantly associated with poor survival in metastatic gastric cancer patients.

Recently, Lee *et al* (2007) published a multivariate analysis on 1445 gastric cancer patients undergoing first-line chemotherapy. No previous gastrectomy, albumin <3.6 g per 100 ml, alkaline phosphatase >85 U l^−1^, PS⩾2, bone metastases, and ascites were the main clinical parameters associated with poor survival. The authors used these factors to develop a prognostic model to predict survival by categorising patients into three risk groups: low, intermediate, and high, with corresponding median survival times of 12.5, 7, 2.7 months, respectively (*P*<0.001).

Even if no effective second-line chemotherapy has been established yet, in this setting several phase II trials were published during the last decades (Schöffski 2002; Wilson *et al*, 2005; Sastre *et al*, 2006). These studies suggest response rates similar to those achieved in other cancers, such as colorectal, lung, ovarian cancers (Schöffski 2002; Colombo *et al*, 2006; Goldberg *et al*, 2007; Vansteenkiste, 2007). However, we still lack of phase III trials comparing second-line chemotherapy *vs* best supportive care.

In this report, we used the independent prognostic factors to define three different risk groups of patients, low-risk group, patients with no prognostic factor; intermediate-risk group, patients with one or two negative prognostic factors; high-risk group, patients with three to five negative prognostic factors, with median survival time of 12.7, 7.1, and 3.3 months, respectively. The benefit of each risk group is quite similar to that reported in the other series including patients treated with first-line chemotherapy (Chau *et al*, 2004b; Lee *et al*, 2007). Therefore, it is plausible that patients with no or a limited number of poor prognostic factors may derive the same benefit from second-line chemotherapy as patients treated with first-line chemotherapy.

This analysis confirms the importance of PS when receiving second-line treatment (Yoshida *et al*, 2004; Chau *et al*, 2004b; Lee *et al*, 2007). As general conditions of patients with advanced gastric cancer may rapidly deteriorate after first-line chemotherapy, second-line therapy should be limited to patients with good PS. Moreover, consideration of good PS is of high importance not only as it is associated with OS, but also when we consider tolerability of chemotherapy.

Park *et al* (2006) suggested that low baseline haemoglobin level (<10 g l^−1^) is a strong and independent prognostic factor for the outcomes of advanced gastric cancer patients receiving 5-FU-based first-line chemotherapy. Similarly, in our analysis, haemoglobin level was found of prognostic value after uni- and multivariate analysis, while conflicting results were emphasised in other reports considering the presence of anaemia at onset of first-line chemotherapy (Chau *et al*, 2004b; Lee *et al*, 2007).

Serum CEA level is a common preoperative and follow-up marker of gastrointestinal tumours (Holyoke *et al*, 1975; Korenaga *et al*, 1995). Serum CEA is a useful indicator of potential curability in patients who undergo gastrectomy and it provides predictive value in determining tumour stage and prognostic information for patients with potentially resectable gastric cancer (Ikeda *et al*, 1995; Tachibana *et al*, 1998; Kochi *et al*, 2000). Increased CEA was found to be univariate adverse prognostic factor for survival in locally advanced or metastatic gastric cancer patients treated with first-line chemotherapy (Louvet *et al*, 2003). In our series, CEA >50 ng ml^−1^ was a negative prognostic factor and this may reflect the metastatic potential of malignant cells (Hostetter *et al*, 1990).

The presence of peritoneal metastases or ascites are poor independent prognostic factors for gastric cancer patients receiving first-line chemotherapy (Chau *et al*, 2004b; Lee *et al*, 2007). In the present analysis, peritoneal metastasis had some prognostic value at the univariate analysis, but the multivariate analysis failed to show any independent prognostic significance. We cannot rule out a possible interaction of peritoneal disease with other factors, such as PS, number of metastatic sites, or TTP under first-line chemotherapy. Patients with clinically or radiologically gross peritoneal metastases also more frequently have lower PS, poor tumour response to chemotherapy and survival than patients with small amount of peritoneal disease or diagnosed at explorative laparotomy (Sadeghi *et al*, 2000; Chau *et al*, 2004b). Number of metastatic sites of disease was significantly associated with OS, and this confirms what was previously reported in gastrointestinal cancers (Mitry *et al*, 2004; Yoshida *et al*, 2004).

In a small report, TTP under first-line treatment has been suggested as useful selection criteria for second-line chemotherapy (Stahl *et al*, 2005). It was a predictor for patients (*n*=27) who did benefit from the second-line chemotherapy with a median survival from the start of second-line therapy of 10.6 months, compared to 5.1 months for the overall group. For this reason, in our analysis we explored the possible prognostic role of TTP under first-line chemotherapy. Patients with TTP under first-line chemotherapy ⩽6 months and treated with second-line chemotherapy had a median survival time since the starting date of the second-line therapy of 11 months, which represents the best median OS of active first-line triple-agents regimens for metastatic gastric cancer (Wagner *et al*, 2006). Then, patients with rapidly progressing disease are an unfavourable setting of patients more unlikely to take advantage from second-line chemotherapy.

The present analysis has been performed in patients receiving second-line chemotherapy and it cannot account for novel biologic agents. Therefore, any conclusion cannot be generalised for the treatments including cytotoxic drugs and biologic agents. Clinico-pathologic and tumour biologic factors may help to find optimal candidates for additional therapy with targeted agents and cytotoxic drugs, also in the context of clinical trials. This strategy may be crucial to develop new approaches for this lethal disease. Moreover, patients with high-risk factors should be refrained from fruitless cytotoxic therapy, and this group of patients may be the one that should be candidate for non-cytotoxic treatments.

In conclusion, in the absence of a standard regimen, this analysis suggests that some clinical factors, which are easily identifiable, may define some groups of patients with gastric cancer more likely to benefit from a second-line chemotherapy. Phase III randomised trials comparing second-line chemotherapy *vs* best supportive care in patients with advanced gastric cancer are highly warranted. However, we also need to evaluate new agents including the targeted agents for this population incorporating prognostic and predictive markers as a component of trial design.

## Figures and Tables

**Table 1:** Patient characteristics at baseline (*n*=175)

**Characteristics**	**No. of patients**
Age (median age, range)	64 (38–83)
Male/female	116/59
	
*Previous gastrectomy*	
*Performance status (ECOG)*	
0/1/2	53/76/41
	
*Disease status*	
Locally advanced/metastatic	4/171
	
*Site of primary tumour*	
GEJ/cardia	28
Body	66
Antrum	63
Anastomosis	9
Multiple sites	9
	
*Previous gastrectomy*	
Yes/no	136/49
	
*Histology*	
Adenocarcinoma	139
Signet ring cell carcinoma	27
Undifferentiated	9
	
*Grade*	
Well/moderately/poorly differentiated	5/41/102
Not known	27
	
*Lauren classification*	
Intestinal/diffuse	105/70
	
Haemoglobin, g l^−1^ (median, range)	11.5 (7.3–15.6)
Albumin, g per 100 ml (median, range)	3.5 (1.8–4.9)
CEA, ng ml^−1^(median, range)	50 (1 to >500)
	
*Sites of disease*	
Stomach/local relapse	77
Liver	66
Peritoneum	80
Lymph node	69
	
*Number of metastatic sites*	
1–2	125
3–4	50

ECOG=Eastern Cooperative Oncology Group; GEJ=gastroesophageal junction.

**Table 2:** Results of first-line and second-line chemotherapy (*n*=175)

	**No. of patients**
First-line chemotherapy	
*Treatment*	
5-FU-based	13
5FU/cisplatin-based	141
5-FU/oxaliplatin-based	21
	
*Response rate*	
CR	12
PR	71
ORR	47.4% (95% CI, 40.0–54.8)
SD	48
PD	44
TTP, median (range)	6 months (1–68)
	
Second-line chemotherapy	
*Treatment*	
5-FU-based	47
5-FU/CDDP-based	21
5-FU/oxaliplatin-based	31
5-FU/irinotecan-based	51
5-FU/taxane	25
	
*Response rate*	
CR	3
PR	25
ORR	16.0% (95% CI, 10.6–21.4)
SD	59
PD	88

5-FU=5-fluorouracil; CI=confidence interval; CR=complete response; ORR=overall response rate (according to RECIST); PD=progressive disease; PR=partial response; SD=stable disease; Taxane=docetaxel, paclitaxel; TTP=time-to-progression.

**Table 3:** Univariate analysis (*n*=175)

**Variable**	**MST (months)**	**1-year survival (%)**	***P*-value**
*Age*			
⩽64	6.0	23.2	0.396
>64	6.0	18.2	
			
*Sex*			
Male	6.3	18.7	0.967
Female	5.8	24.6	
			
*Performance status (ECOG)*			
0–1	7.5	25.3	<0.001
2	2.5	8.1	
			
*Loss of weight* >*5*%			
Yes	5.0	14.7	0.003
No	7.5	24.3	
			
*Haemoglobin*			
⩽11.5 g l^−1^	5.0	14.2	0.002
>11.5 g l^−1^	7.8	28.8	
			
*Albumin*			
⩽3.5 g per 100 ml	5.5	19.5	0.08
>3.5 g per 100 ml	6.8	26.7	
			
*CEA*			
⩽50 ng ml^−1^	7.0	25.2	0.002
>50 ng ml^−1^	5.0	3.8	
			
*Previous gastrectomy*			
Yes	6.6	21.7	0.23
No	4.1	16.6	
			
*Lauren classification*			
Intestinal	6.5	19.4	0.791
Diffuse	5.5	22.2	
			
*Grade*			
Well to moderate	6.5	18.4	0.447
Poor	6.2	22.2	
			
*Site of primary*			
GEJ to cardia	6.5	25.4	0.365
Body to antrum	6.0	19.7	
			
*Liver metastasis*			
Yes	5.5	13.9	0.194
No	6.6	24.8	
			
*Peritoneal metastasis*			
Yes	5.0	14.6	0.005
No	7.3	25.5	
			
*Number of metastatic sites*			
1–2	7.2	26.2	<0.001
3–4	3.8	6.2	
			
*First-line chemotherapy*			
5-FU-based	4.5	0.0	0.021
5FU/platinum-based	6.1	19.1	
			
*Response to first-line chemotherapy*			
Yes	7.0	24.3	0.297
No	5.5	16.6	
			
*TTP of first-line chemotherapy⩽6 mos*			
Yes	4.8	9.1	<0.001
No	11.0	36.6	
			
*Year*			
1995–2000	5.5	3.8	0.128
2001–2006	7.2	4.9	

5-FU=5-fluorouracil; ECOG=Eastern Cooperative Oncology Group; GEJ=gastroesophageal junction; mos=months; MST=median survival time; Platinum=cisplatin, ovaliplatin; TTP=time-to-progression.

**Table 4:** Multivariate analysis (*n*=175)

**Variable**	**HR**	**95% CI**	***P*-value**
ECOG performance status 2	1.79	1.16–2.77	0.008
Loss of weight >5%	1.05	0.73–1.53	0.77
Haemoglobin ⩽11.5 g l^−1^	1.48	1.06–2.05	0.019
CEA>50 ng ml^−1^	1.86	1.21–2.88	0.004
Peritoneal metastasis	1.16	0.80–1.68	0.41
Number of metastatic sites 3–4	1.72	1.16–2.53	0.006
5-FU-based first-line chemotherapy	1.97	0.86–3.74	0.0733
TTP of the first-line chemotherapy ⩽6 months	1.97	1.39–2.80	<0.0001

5-FU=5-fluorouracil; CI=confidence interval; ECOG=Eastern Cooperative Oncology Group; HR=hazard ratio; TTP=time-to-progression.
